# Catalytic Oxidation of Naphthalene and Polycyclic Arenes by Iron(III) TAML/H_2_O_2_ in Water Aiming at Their Efficient Removal from Aqua Natural Systems

**DOI:** 10.1002/chem.202500450

**Published:** 2025-07-09

**Authors:** Parameswar Pal, Chimezie Anyakora, Alexander D. Ryabov, Terrence J. Collins

**Affiliations:** ^1^ Institute for Green Science Department of Chemistry Carnegie Mellon University 4400 Fifth Avenue Pittsburgh PA 15213 USA; ^2^ Department Chemistry School of Science and Technology Pan‐Atlantic University Main Campus Km 52, Lekki‐Epe Expressway (Near Eleko Beach Junction) Ibeju‐Lekki Lagos Nigeria

**Keywords:** hydrogen peroxide, naphthalene, polyarenes, TAML, water treatment

## Abstract

The electron transfer from naphthalene at an oxidized iron TAML species (TAML = *t*etra*a*mido *m*acrocyclic *l*igand) is a key step of its environmentally relevant deep degradation by hydrogen peroxide in water leading first to naphthoquinones which are further converted to smaller fragments. Other polycyclic arenes are also oxidized, often faster than naphthalene consistent with their lower ionization potentials than that of naphthalene.

## Graphical abstract

Fe‐TAML in the presence of hydrogen peroxide efficiently catalyzes the oxidative degradation of naphthalene in *aqueous* medium of neutral pH. Products are identified and key mechanistic features are discussed.

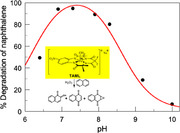

In 2018 Hedman, Hodgson, Solomon, Cho, and others described the homogeneous oxidation of naphthalene by a Mn^IV^ bis(hydroxo) complex.^[^ ^1^ ^]^ The study had a strong environmental flavor because industrial development based on fossil fuels has introduced myriad polycyclic arenes which are iconic of the patterns and perilous ecospherical insults that can result.^[^ ^2^ ^]^ Threats from polyarenes were recognized long time ago;^[^ ^3^ ^]^ polyarenes are carcinogenic,^[^ ^4^ ^]^ hydrophobic, they bioaccumulate up the food chain^[^ ^5^ ^]^ leading to well‐earned listings by the United States Environmental Protection Agency (US EPA) as priority pollutants.^[^ ^6^ ^]^ There was just a single stumbling stone in the 2018 work^[^ ^1^ ^]^ — the transformations described occurred in an organic solvent which bans the process from using in the real world for solving ecological tasks. The similarly efficient oxidation of naphthalene should occur in water and here we report on the iron TAML system which is capable of efficient catalytic oxidation of polycyclic arenes in neutral aqueous solutions.

Oxidative degradation of environmental pollutants by oxidizing metal species should be catalytic. Otherwise, substantial loadings of metal oxidants in the ecosphere will be required. The primary oxidant should be inexpensive and ecofriendly. Such are hydrogen peroxide and dioxygen. Iron(III) TAML activators of peroxides (Figure 1) fit these requirements because (i) the central metal is a nature‐friendly element, viz. iron, (ii) the activators operate in aqueous medium using (iii) H_2_O_2_, and (iv) they have proven their potential in various ecologically relevant processes.^[^ ^7^, ^8^, ^9^, ^10^ ^]^ Here we show that iron(III) TAMLs catalyze deep degradation of naphthalene and other polycyclic aromatic hydrocarbons (PAHs).

**Figure 1:**
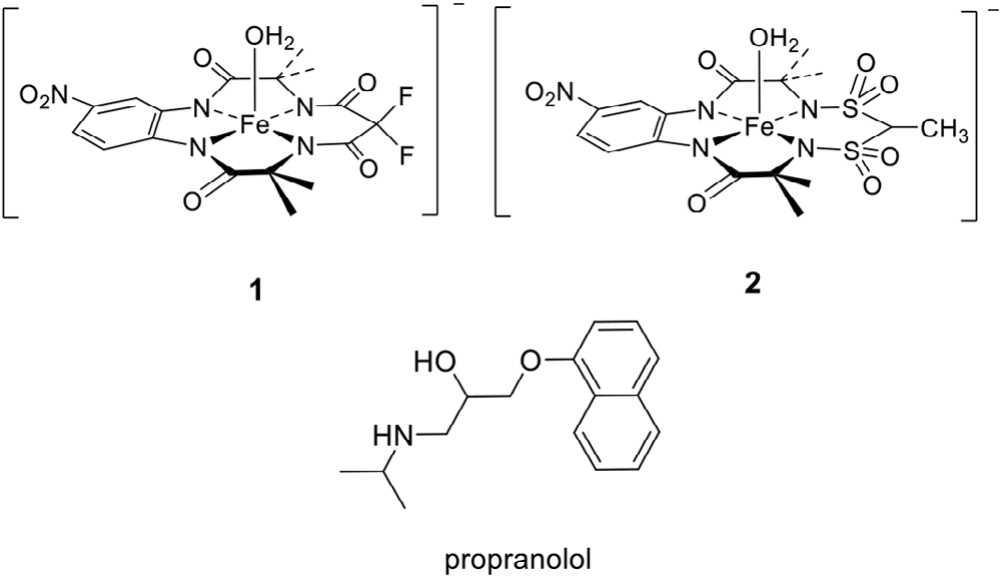
The TAML activators used in this study plus structure of propranolol.

The oxidative degradation of naphthalene (7.8 × 10^−8^ M, 10 ppb) by H_2_O_2_ (2.9 × 10^−4^ M, 10 ppm) catalyzed by **1** (1.2 × 10^−7^ M, 60 ppb) or **2** (2.8 × 10^−7^ M, 160 ppb) was explored at neutral pH and ambient temperature. Low concentrations of all ingredients were selected due to environmental needs to degrade particularly low doses of naphthalene by minimal amounts of H_2_O_2_/catalyst to reduce their potential harmful effects on aquatic species.^[^ ^11^ ^]^ Therefore, the catalytic activity of **1** and **2** should be high to ensure efficient degradation of naphthalene. The results in Figure 2a show that the TAML catalysts are efficient since 91% of naphthalene are degraded in a matter of 2.5 hours whereas H_2_O_2_ alone is ineffective. The 100% efficacy was not achieved due to the catalyst inactivation during the catalytic cycle^[^ ^12^ ^]^ since addition of the second aliquot of **1** increases the conversion up to 96%.

**Figure 2:**
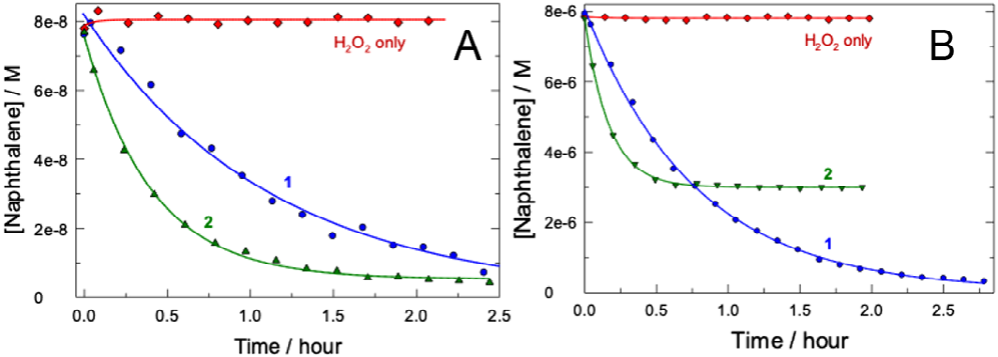
a) Removing naphthalene (7.8 × 10^−8^ M) by H_2_O_2_ (2.9 × 10^−4^ M) in the presence of **1** (1.2 × 10^−7^ M) and **2** (2.8 × 10^−7^ M). b) Removing of 8.0 × 10^−6^ M naphthalene by 0.01 M H_2_O_2_ in the presence of 1.6 × 10^−7^ M **1** or 8.0 × 10^−8^ M **2**. Conditions: pH 6.9 (0.01 M phosphate) and 25 °C. Lines are drawn for emphasis only.

Tetra‐amido TAML **1** overperforms bis‐amido bis‐sulfonamido catalyst **2** particularly at 100‐fold higher concentrations of naphthalene (Figure 2b). The reaction is far from completion in the case of **2** due to the documented operational instability of **2**.^[^ ^12^, ^13^ ^]^ Correspondingly, most of the data reported here were collected using more aggressive to naphthalene catalyst **1**.

The pH effects of the catalytic activity of **1** and on amounts of naphthalene removed in 1 hour are shown in Figure 3. Real world environmental applications of water sanitizers require their highest reactivity at neutral pH. In the pH range of 6.4 − 10.0 the maximal rate and the highest conversion of naphthalene were detected at pH 7.3 (Figure 3).

**Figure 3:**
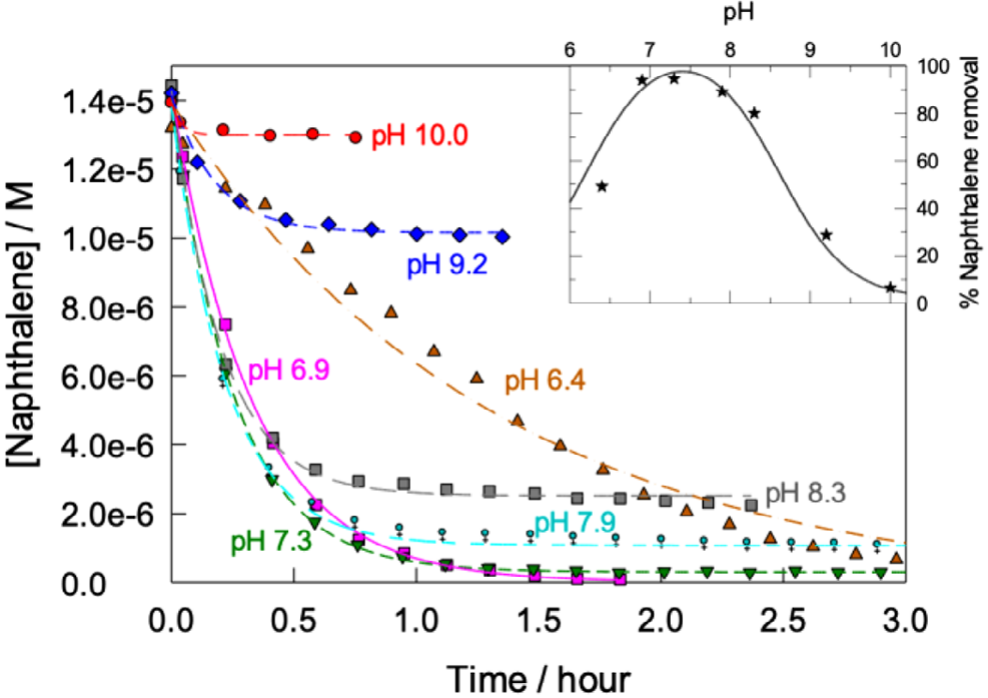
Degradation of naphthalene (1.4 × 10^−5^ M) at different pH in 0.01 M phosphate (pH 6.4 − 8.3), or carbonate buffers (pH 9.2 ^–^ 10.0). Conditions: [**1**] 1.6 × 10^−7^ M, [H_2_O_2_] 0.02 M, 25 °C. Lines were drawn for emphasis only. Inset shows amounts of naphthalene removed in 1 hour at different pH.

Up‐to‐date strategies of decomposition of environmental pollutants call for detailing fragments of their degradation because the fragments may be more toxic than their predecessors.^[^ ^14^ ^]^ The naphthalene pieces were assigned by the HPLC technique using significantly higher naphthalene concentration compared to that in Figure 2. A relatively low concentration of H_2_O_2_ was also used for minimizing further oxidation of naphthalene fragments. Though under such conditions the naphthalene conversion is just 40%, both sensitivity and time scale are optimal for analytical purposes. The HPLC spectra reflecting the catalytic oxidation of naphthalene (Figure S1, Supporting Information) contain several major peaks with retention times lower than that of naphthalene (retention time 9.3 minutes). They arise from 1,2‐naphthoquinone (3.3 min), 1,4‐naphthoquinone (4.0 min), and 2,3‐dihydro‐2,3‐epoxy‐1,4‐naphthoquinone (3.8 minutes). The dynamics of generation of the major fragments alongside the naphthalene collapse is illustrated in Figure 4. The inflection point at the 2,3‐dihydro‐2,3‐epoxy‐1,4‐naphthoquinone versus time profile and maximal concentration of 1,4‐naphthoquinone are both observed at practically same time suggesting that the former is produced from the latter. The formation of both was anticipated in light of our recent results on the catalytic degradation of propranolol (1‐(naphthalen‐1‐yloxy)‐3‐(isopropylamino)propan‐2‐ol, Figure 1) by **1**/H_2_O_2_, where 1,2‐naphthoquinone was not detected.^[^ ^15^ ^]^ 2,3‐Dihydro‐2,3‐epoxy‐1,4‐naphthoquinone is further degraded by H_2_O_2_ in parallel noncatalytic and **1**‐catalyzed pathways (Figure S2).

**Figure 4:**
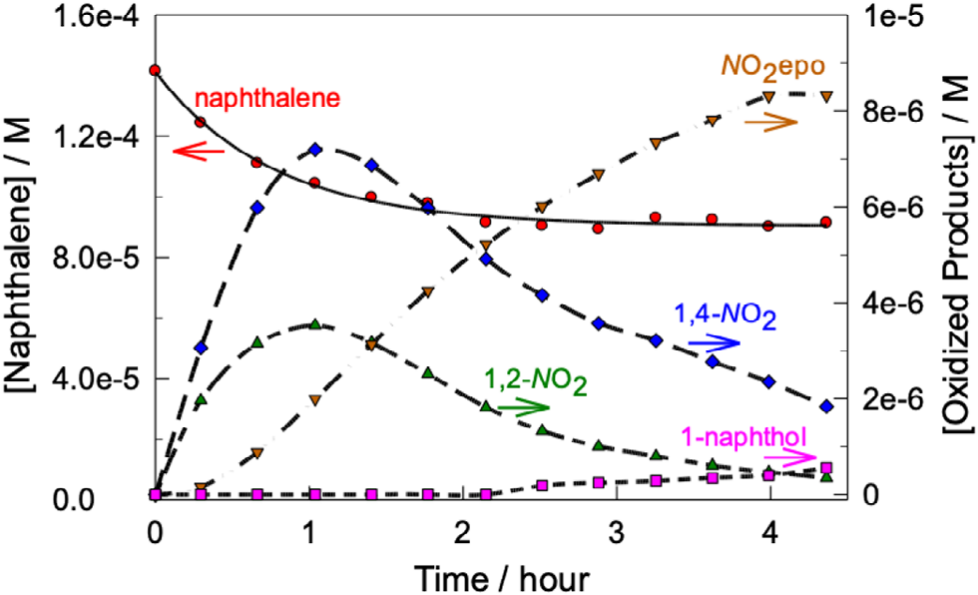
Dynamics of generation of 1,2‐naphthoquinone, 1,4‐naphthoquinone, 2,3‐dihydro‐2,3‐epoxy‐1,4‐naphthoquinone, and 1‐naphthol alongside with naphthalene collapse. Conditions: [naphthalene] 1.4 × 10^−4^ M, 3.2 × 10^−7^ M, [H_2_O_2_] 6.0 × 10^−3^ M, pH 6.9 (0.01 M phosphate), 25 °C. Abbreviations: 1,2‐*N*O_2_, 1,2‐naphthoquinone;1,4‐*N*O_2_, 1,4‐naphthoquinone; *N*O_2_epo, 2,3‐dihydro‐2,3‐epoxy‐1,4‐naphthoquinone.

Naphthoquinones are most probably produced from the corresponding dihydroxynaphthalenes and H_2_O_2_^[^ ^14^ ^]^ though naphthols should be their initial precursors. Interestingly, the 1‐napththol formation in Figure 4 is detectable after ca. 2 hours when the naphthalene degradation becomes very slow.

It was natural to anticipate the naphthol appearance at early stages of the reaction prior to 1,2‐ and 1,4‐naphthoquinone peaks, that is, in a matter of ca. 10–30 minutes. The naphthol invisibility is due to its much higher reactivity compared to naphthalene (vide infra). Less than 25 minutes are needed for complete elimination of 1‐naphthol, the products detected by HPLC being the same as for naphthalene, viz. 1,2‐ and 1,4‐naphthoquinone plus 2,3‐dihydro‐2,3‐epoxy‐1,4‐naphthoquinone, Figures 5 and S3. This observation suggests that the formation of 1‐naphthol at later stages of naphthalene degradation when **1** is practically inactive^[^ ^12^ ^]^ is due to secondary pathways without TAML participation. Such processes have been previously described.^[^ ^16^, ^17^ ^]^ Correspondingly, addition of the second aliquot of **1** results in the rapid disappearance of 1‐naphthol (Figure S4). There are minor overlapping peaks with retention times in the range of 2.5–3.0 minutes (Figure S1). These could be due to 2‐hydroxynaphthalene‐1,4‐dione produced by **1**/H_2_O_2_ from 1,4‐naphthoquinone^[^ ^15^ ^]^ and *o*‐carboxycinnamic acid which is the product of oxidation of 1,2‐naphthoquinone by H_2_O_2_.^[^ ^18^ ^]^ It has been previously confirmed by ion chromatography that the naphthalene fragments are finally converted to phthalic acid, small aliphatic acids, and acetone (traced by ^1^H NMR).^[^ ^15^ ^]^ These facts finalize Scheme 1 which shows a spectrum of products produced in the naphthalene/**1**/H_2_O_2_ system.

**Figure 5:**
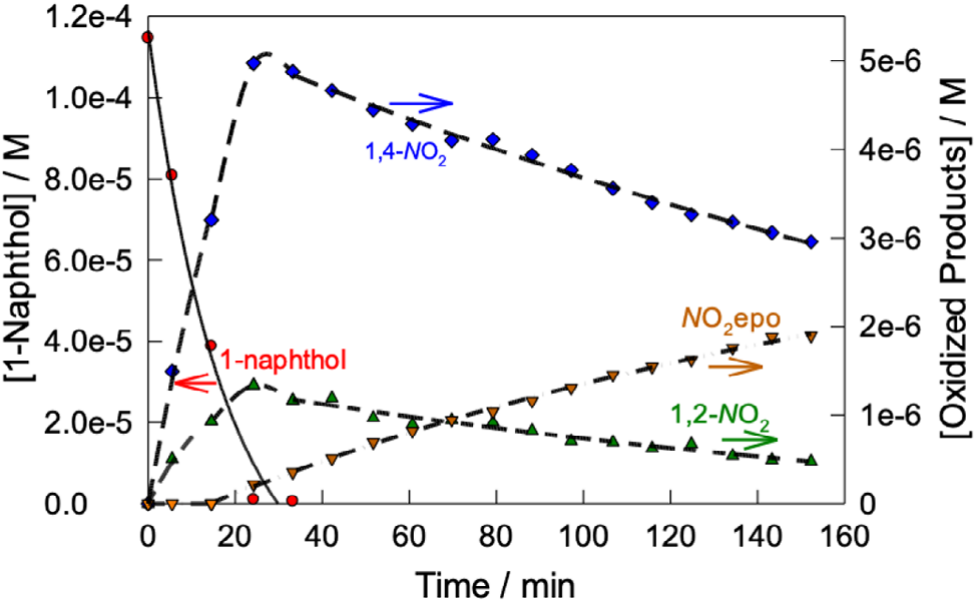
Dynamics of generation of 1,2‐ and 1,4‐naphthoquinone, 2,3‐dihydro‐2,3‐epoxy‐1,4‐naphthoquinone from 1‐naphthol. Conditions: [1‐naphthol] 1.1 × 10^−4^ M, 1.6 × 10^−7^ M, [H_2_O_2_] 3.0 × 10^−3^ M, pH 6.9 (0.01 M phosphate), 25 °C. Abbreviations: 1,2‐*N*O_2_, 1,2‐naphthoquinone; 1,4‐*N*O_2_, 1,4‐naphthoquinone; *N*O_2_epo, 2,3‐dihydro‐2,3‐epoxy‐1,4‐naphthoquinone.

**Scheme 1:**
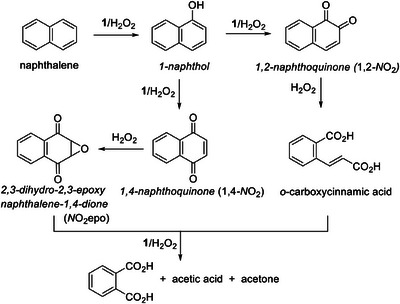
Detected (italicized) and postulated products of the degradation of naphthalene by **1**/H_2_O_2_ in neutral *aqueous* solution.

Higher efficacy of the TAML system versus other previously reported routines for the naphthalene decontamination is straightforward. In fact, Cl_2_ (6.6 × 10^−5^ M, 4.7 ppm) does not degrade naphthalene at 5 nM (0.64 ppb).^[^ ^19^ ^]^ The effect of Cl_2_ is observable at 5 × 10^4^ ppb naphthalene (400 µM) in *aqueous* media containing 1% methanol and high [Cl_2_] of 850 ppm (0.011 M).^[^ ^20^ ^]^ Facile oxidation of 44 ppb naphthalene (3.4 × 10^−6^ M) in water has been reported to occur by 2.3 equivalents of ozone (0.4 ppm) at 1 °C.^[^ ^21^ ^]^ Ozone converts naphthalene to phthalaldehyde and phthalaldehydic acid.^[^ ^22^ ^]^ These products, which are more toxic (LC_50_ at 96 hours for phthalaldehyde is 0.07 mg L^−1^ based on study on freshwater fish)^[^ ^23^ ^]^ than naphthalene (LC_50_ at 96 hours for naphthalene is 8.4 mg L^−1^ based on study on zebrafish, *Danio rerio*),^[^ ^24^ ^]^ have not been detected in the TAML system.

Polycyclic arenes other than naphthalene (Figure 6) are also degradable by **1**/H_2_O_2_. Those with sufficient solubility in water, viz, fluorene, acenaphthene, and phenanthrene, were tested first (Figure 7). Interestingly, some of them are more reactive than naphthalene and their > 75% degradation is achieved in less than 13 minutes at pH 6.9 in the presence of (0.02–0.04) M H_2_O_2_ and (8.0–32) × 10^−8^ M **1**. Major products detected by HPLC for fluorene were 9‐hydroxyfluorene and 9‐fluorenone; acenaphthene gave 1‐acenaphthenol and acenaphthoquinone (see Figures S5 and S6 for details).

**Figure 6:**
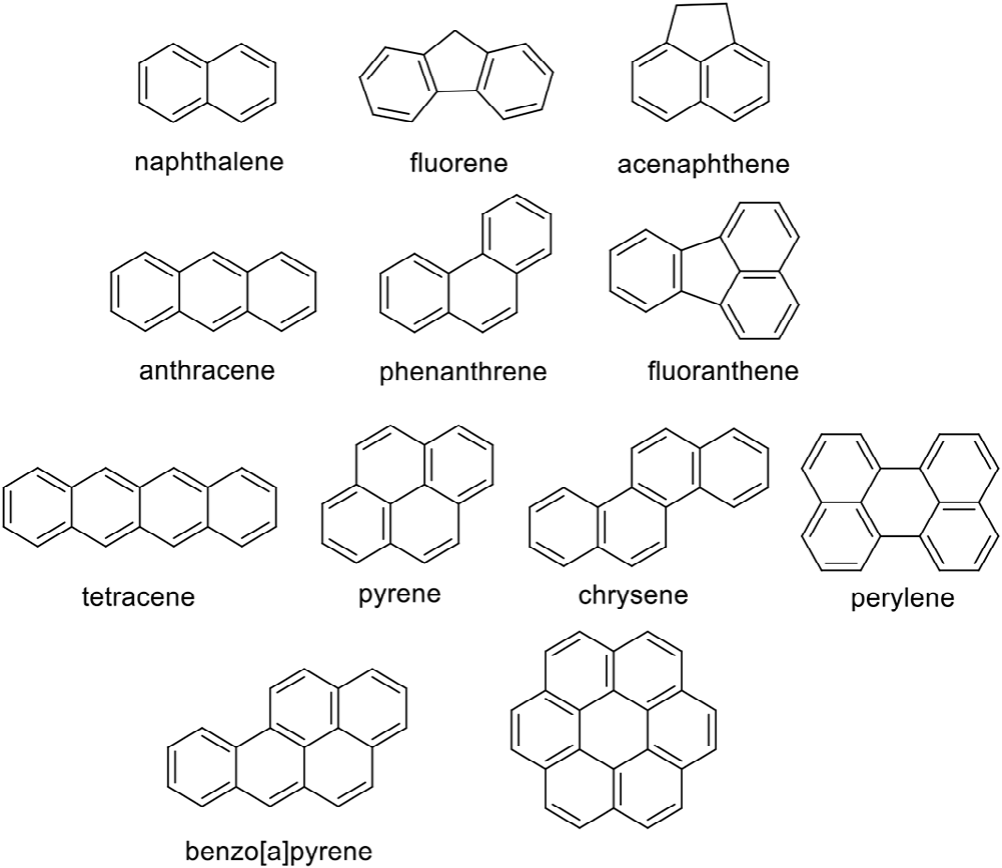
Polycyclic arenes explored in this work.

**Figure 7:**
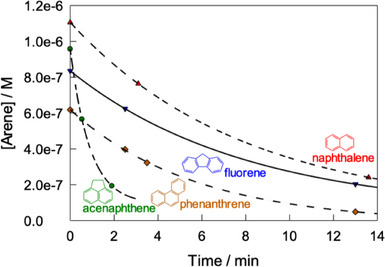
Degradation of naphthalene, fluorene, acenaphthene, and phenanthrene by **1**/H_2_O_2_ at pH 6.9 and 25 °C. Other conditions: [naphthalene] 1.1 × 10^−6^ M, 1.6 × 10^−7^ M, [H_2_O_2_] 0.04 M; [fluorene] 8.4 × 10^−7^ M, 1.6 × 10^−7^ M, [H_2_O_2_] 0.02 M; [phenanthrene] 6.2 × 10^−7^ M, 3.2 × 10^−7^ M, [H_2_O_2_] 0.04 M; [acenaphthene] 9.6 × 10^−7^ M, 8.0 × 10^−8^ M, [H_2_O_2_] 0.02 M; pH 6.9 (0.01 M phosphate), 25 °C. Lines were drawn for emphasis only.

Low solubility of other polyarenes forced us to explore their behavior in the presence of 5–45% (*v/v*) acetonitrile added to the buffered water. The results collected in Table 1 confirm high potential of **1**/H_2_O_2_ in destroying polyaromatic compounds.

**Table 1:** Oxidative degradation of polyaromatic compounds by **1**/H_2_O_2_ in water in the presence of MeCN. Conditions: [**1**] (8.0–16.0) × 10^−8^ M, [H_2_O_2_] 0.01–0.04 M, [arene] (0.12–1.60) × 10^−5^ M (see Supporting Information for exact concentrations), pH 6.9, 25 °C.

Entry	Polyarene	MeCN [%]	Time [min]	Degradation [%]
1	Benzo[*a*]pyrene	5	11.7	97.6
2	Pyrene	5	12	97.9
3	Tetracene	15	142	91.2
4	Anthracene	5	3.9	83.9
5	Chrysene	5	172.7	41.2
6	Fluoranthene	5	45.4	48.1
7	Perylene	15	11.7	85.3
8	Coronene	45	102.2	26.7

The mechanistic disclosure of naphthalene oxidation by the Mn^IV^ species^[^ ^1^ ^]^ was very thorough. Matching products are formed in the Mn^IV^ and TAML systems. Perhaps a deep new search for the TAML reaction mechanism would not provide new insights. Sufficient is just to confirm similarities of the key mechanistic features which support the rate‐limiting electron transfer.^[^ ^1^ ^]^ Peroxides transform iron(III) to iron(IV) TAML species in pure water ^[^ ^7^ ^]^ and this makes the iron and manganese systems even closer to each other. The limited kinetic data were obtained by following initial rate of disappearance of naphthalenes by HPLC under the conditions when the oxidation of iron(III) by H_2_O_2_ is fast and the rate‐limiting step is the interaction of catalytically active TAML species with naphthalenes.^[^ ^12^ ^]^ The kinetic isotope effect *k*_H_/*k*_D_ of 1.0 was previously obtained with naphthalene‐*d*_8_.^[^ ^1^ ^]^ The value of *k*_H_/*k*_D_ of 1.3 ± 0.1 was found for the **1**/H_2_O_2_ system (Figure S7) consistent with a slow electron transfer at an oxidized iron species produced from iron(III) and H_2_O_2_.

As in the Mn^IV^ case, the reactivity of **1** was tested with 1‐substituted naphthalenes and a similar trend was established with the Hammett ρ value of −1.1 versus σ^+^ (Figure 8), that is, electron‐donating groups increase the oxidation rate. The confirmed 70‐fold higher reactivity of 1‐naphthol versus naphthalene accounts for why 1‐naphthol has not been detected at early stages of naphthalene degradation (Figure 4).

**Figure 8:**
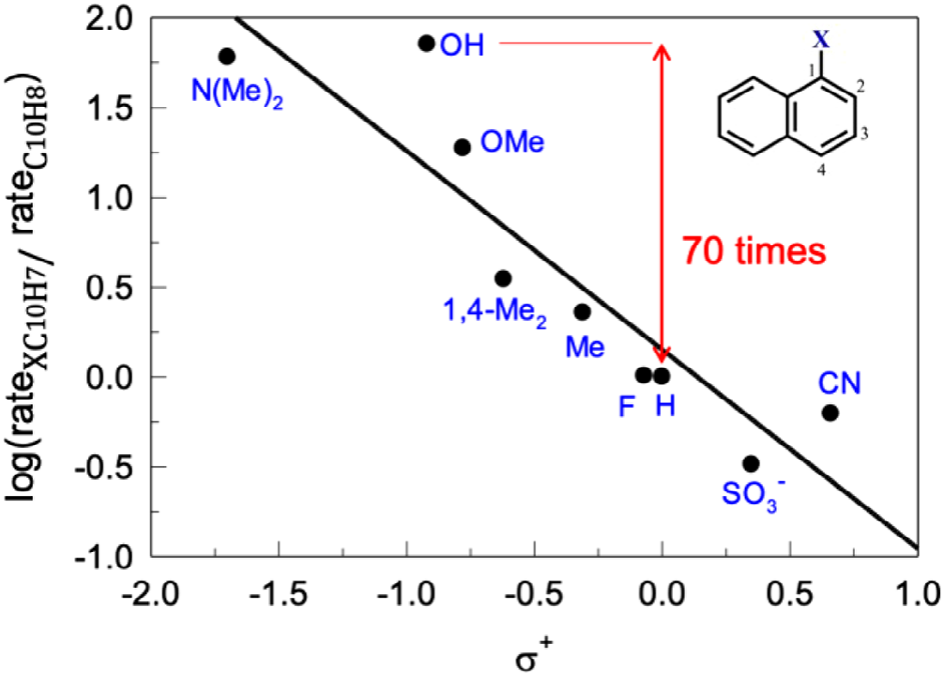
Hammett plot for the relative initial rates of oxidation of 1‐substituted naphthalenes by **1**/H_2_O_2_ versus σ^+^ also showing higher rate of oxidation of 1‐naphthol compared to naphthalene. Conditions: [naphthalenes] 9.0 × 10^−6^ M, 8.0 × 10^−8^ M, [H_2_O_2_] 0.01 M, pH 6.9 (0.01 M phosphate), and 25 °C.

When the substituent effect was analyzed as before,^[^ ^1^ ^]^ viz. the rates were correlated with the oxidation potentials of naphthalenes, a negative slope of −2.7 (Figure S8) was found to be compared with the value of −3.7 reported by Jeong, et al.^[^ ^1^ ^]^ All similarities mentioned above agree with the close similarity of mechanisms of naphthalene oxidation in both systems. The rate‐limiting electron transfer generates naphthalene radical‐cation. The latter leads to 1‐naphthol. Mechanistically similar second hydroxylation of 1‐naphthol produces 1,2‐ and 1,4‐dihydroxynaphthalenes which transform to the corresponding naphthoquinones. The overall TAML mechanism (Scheme S1 of Supporting Information) is practically indistinguishable from that shown in Scheme 1 elsewhere.^[^ ^1^ ^]^

In conclusion, the facile oxidation of 10 ppb of naphthalene using 10 ppm H_2_O_2_ with 1.2 × 10^−7^ M **1** at neutral pH inside a small vial may be considered as a small‐scale duplication of the TAML catalyzed degradation of polyarenes in a futuristic large‐scale water treatment facility. A rate‐determining electron transfer from naphthalene leading to the major products as naphthoquinones agrees with the previous investigation of the oxidation of naphthalene by high‐valent transition metal oxo species. Arenes other than naphthalene are also prone to oxidative degradation by TAML in *aqueous* media.

## Supporting Information

The authors have cited additional references in the Supporting Information.^[^ ^25^, ^26^, ^27^, ^28^, ^29^, ^30^, ^31^, ^32^, ^33^ ^]^

## Conflict of Interest

The authors declare no conflict of interest.

## Supporting information



Supporting Information

## Data Availability

Data sharing is not applicable to this article as no new data were created or analyzed in this study.
