# Supplementary material for: Catalytic Oxidation of Naphthalene and Polycyclic Arenes by Iron(III) TAML/H2O2 in Water Aiming at Their Efficient Removal from Aqua Natural Systems
Source: Chemistry. 2025 Jul 9;31(43):e202500450. doi: 10.1002/chem.202500450 (PMC12319346; doi:10.1002/chem.202500450)
Supplement: Supplementary file 1 — Supporting Information [file CHEM-31-e202500450-s001.pdf]

Supporting Information

Catalytic Oxidation of Naphthalene and Polycyclic Arenes by Iron(III) TAML/H_2O_2 in Water Aiming at Their Efficient Removal from Aqua Natural Systems

Parameswar Pal,[a] Chimezie Anyakora,[b] Alexander D. Ryabov,*[a] Terrence J. Collins*[a]

[a] Institute for Green Science, Department of Chemistry, Carnegie Mellon University, 4400 Fifth Avenue, Pittsburgh, PA, 15213, USA

[b] Department Chemistry, School of Science and Technology, Pan-Atlantic University, Main Campus Km 52, Lekki-Epe Expressway (Near Eleko Beach Junction), Ibeju-Lekki, Lagos, Nigeria

*Correspondence to: ryabov@andrew.cmu.edu; tc1u@andrew.cmu.edu

Table of Contents

Materials. 3

Instrumentation. 3

Quantification of naphthalene. 4

Sample Preparation. 4

Oxidation of naphthalene. 4

Experimental conditions for arenes other than naphthalene. 5

Table S1. 5

Figure S1. 6

Figure S2. 6

Figure S3. 7

Figure S4. 7

Figure S5. 8

Figure S6. 9

Figure S7. 10

Figure S8. 10

Figure S9. 11

Scheme S1 11

Experimental Details

Materials

Naphthalene (99.0%), naphthalene-d_8 (99.0%), and 1,4-naphthoquinone (≥ 96.5%), fluoranthene (99%), fluorene (98%), acenaphthene (99%), 1-naphthol (reagent plus ≥99%) were supplied by Sigma-Aldrich. H_2O_2 (30%) was purchased from Supelco. 1,2-naphthoquinone (97%), anthracene (99%) were received from Acros Organics. Potassium dihydrogen phosphate (for HPLC), sodium carbonate (anhydrous), *N*,*N*-dimethyl-1-naphthyl amine (99%), and HPLC vials were purchased from VWR chemicals. Potassium monohydrogen phosphate (anhydrous), sodium bicarbonate, *o*-phosphoric acid (85%), standard buffer solutions for the calibration of pH meter, HPLC grade water and HPLC grade acetonitrile were obtained from Fischer chemical. **1** was obtained from old TAML stocks of the Institute for Green Science (IGS).[25] **2** was supplied by Sudoc, LLC.[26] 1-Methoxynaphthalene (98+%), naphthalene-1-carbonitrile (95%), 1-fluoronaphthalene (98%), phenanthrene (97%), pyrene (98%), coronene (95%), perylene (99+%) were procured from ThermoFisher scientific. 1-Methylnaphthalene (96%) was purchased from Beantown chemical. Naphthalene-1-sulfonic acid sodium salt (98%) was obtained from Alfa Aesar. Phthalaldehyde (for HPLC labeling), phthalaldehydic acid (>98%), chrysene (>98%), tetracene (>97%), benzo[*a*]pyrene (>95.0%), 1,4-dimethylnaphthalene (>96%) were purchased from Tokyo Chemical Industry (TCI).

Instrumentation

For the detection and quantification of naphthalene, a Shimadzu high performance liquid chromatography (HPLC) system (SIL-20AC autosampler, LC-20AB pump, CTO-20A column over, and SPD-M20A photo diode array (PDA) detector, Lab Solutions (data software) was used. A Kinetex 5 μm EVO C18 100 Å LC column (100 × 4.6 mm) and an isocratic method with solvent system of 10 mM pH 3 phosphate buffer and acetonitrile with a flow rate of 0.5 mL/min was utilized to elute naphthalene. The temperature of the LC column was 35 ^{o}C. The HPLC system was also associated with a temperature-controlled HPLC-vial holder, which permitted collection of kinetic data. The automated integral feature in the LabSolutions software generated peak areas of the eluted peaks. UV-Vis data were collected using an Agilent Cary 3500 Multicell

UV-Vis spectrophotometer. pH of solutions was controlled by an Accumet Basic AB15 pH meter. The pH meter was calibrated daily with the standard buffers of pH 4, 7, and 10.

Quantification of naphthalene

This was done at the wavelength of maximum absorption (220 nm). The volume injected by the autosampler into the LC column was in the range of 10 – 60 μL. The ratios of two eluents for the isocratic method were varied from 1.5 to 0.67. The eluent system, 60:40 acetonitrile : pH 3 phosphate buffer was usually used for the rate measurements at a shorter retention time (~ 6 min). When the goal was to detect the products of naphthalene oxidation, higher amounts of the organic component were used for better accuracy. The concentration of naphthalene was calculated using the linear calibration curve as in Figure S9.

Sample Preparation

To prepare 10 mM buffer solution of pH 6 – 8.0, appropriate amounts of KH_2PO_4 and K_2HPO_4 were added in HPLC grade water. Solutions of pH 3 were made using H_3PO_4 and KH_2PO_4. Sodium bicarbonate and sodium carbonate were used to prepare buffers between pH 9 and 10. 10 mM borate buffer was prepared using boric acid and sodium hydroxide between pH 8 and 9. Stock solutions of **1** and **2** were prepared by dissolving solid TAML in HPLC grade water. *Aqueous* stock solutions of naphthalene were prepared by adding 2 mg of naphthalene to 100 mL buffer of desired pH in a glass jar with overnight stirring. The concentration of the resultant stock solution of naphthalene was determined by UV-Vis at 219 nm using the extinction coefficient of 4.9×10^4 M^{-1} cm^{-1}.[27] The concentration of H_2O_2 was controlled by UV-Vis at 230 nm as reported elsewhere.[28]

Oxidation of naphthalene

Aliquots of stock solutions of naphthalene and TAML were pipetted into a HPLC vial to minimize the headspace limiting possible volatilization of naphthalene as suggested elsewhere.[29] The HPLC vials were kept closed with PTFE (polytetrafluoroethylene) caps to reduce the adsorption.[30] The oxidation was started by adding 10 μL of a stock solution of hydrogen peroxide followed by vigorous shaking of the vial.

Experimental conditions for arenes other than naphthalene

Other arenes were analyzed using the same Shimadzu high performance liquid chromatography (HPLC) system. Stock solutions of fluorene, acenaphthene and phenanthrene were prepared using pH 6.9 phosphate buffer, whereas other arenes were dissolved in HPLC-grade acetonitrile. Isocratic methods with 10 mM pH 3 phosphate buffer and acetonitrile were employed. Experimental details are in Table S1. The products of arene oxidation by TAML/H_2O_2 were identified by matching their UV-Vis spectra with those in the literature.[31–33]

Table S1. HPLC methods used to separate different arenes and pertinent reaction conditions used to perform oxidative degradation

Arenes	pH 3 (0.01 M phosphate): acetonitrile (v/v)	Flow rate / mL min^{-1}	Wavelength of detection / nm	[Arene] (M) / [**1**] (M) / [H_2O_2] (M) at pH 6.9, 25 °C
anthracene	35: 65	0.5	251	1.3 ×10^{-5} / 8.0 ×10^{-8} / 0.01
chrysene	35: 65	0.65	267	2.1 ×10^{-6} / 1.6 ×10^{-7} / 0.04
tetracene	35: 65	0.8	274	3.4 ×10^{-6} / 8.0 ×10^{-8} / 0.01
benzo[*a*]pyrene	33: 67	0.85	296	1.9 ×10^{-6} / 1.6 ×10^{-7} / 0.04
pyrene	38: 62	0.65	240	1.6 ×10^{-5} / 1.6 ×10^{-7} / 0.04
coronene	30: 70	1	302	1.2 ×10^{-6} / 1.6 ×10^{-7} / 0.04
fluoranthene	35: 65	0.5	236	4.2 ×10^{-6} / 1.6 ×10^{-7} / 0.04
perylene	33: 67	0.75	251	3.7 ×10^{-6} / 1.6 ×10^{-7} / 0.04

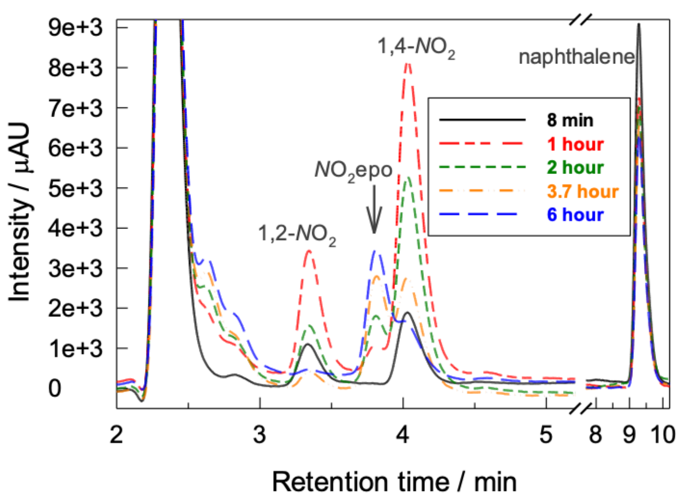

Figure S1. Time-dependent HPLC spectra accompanying naphthalene oxidation by **1**/H_2O_2 with the formation of 1,2-*N*O_2, 1,4-NO_2 and *N*O_2epo. Conditions: [naphthalene] 1.6 $\times 10^{-4}$ M, [**1**] 3.2 $\times 10^{-7}$ M, [H_2O_2] 6.0 $\times 10^{-3}$ M, pH 6.9 (0.01 M phosphate), 25 °C, mobile phase 0.01 M phosphate (pH 3)/acetonitrile (50/50), flow rate 0.5 mLmin^{-1}, wavelength of detection 250 nm.

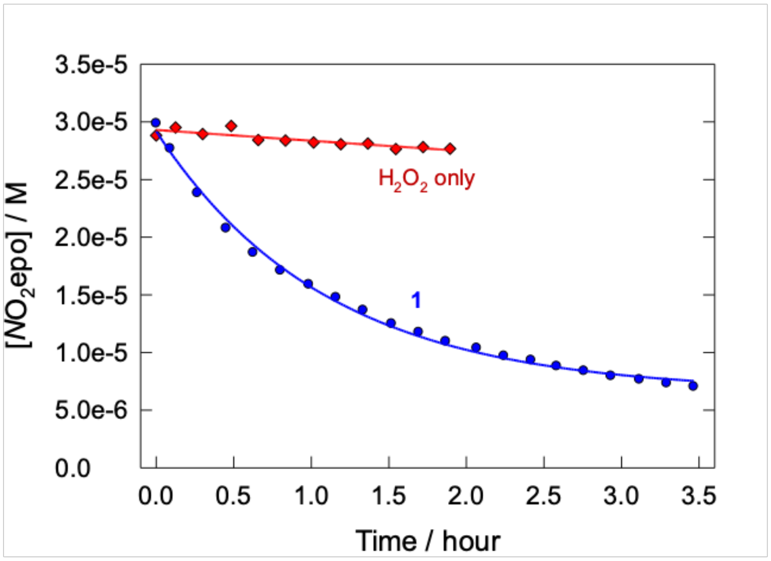

Figure S2. Degradation of 2.9 $\times 10^{-5}$ M 2,3-dihydro-2,3-epoxy-1,4-naphthoquinone (*N*O_2epo) in the presence of 3.2 $\times 10^{-7}$ M **1** and 0.02 M H_2O_2 at pH 6.9 (0.01 M phosphate), 25 °C.

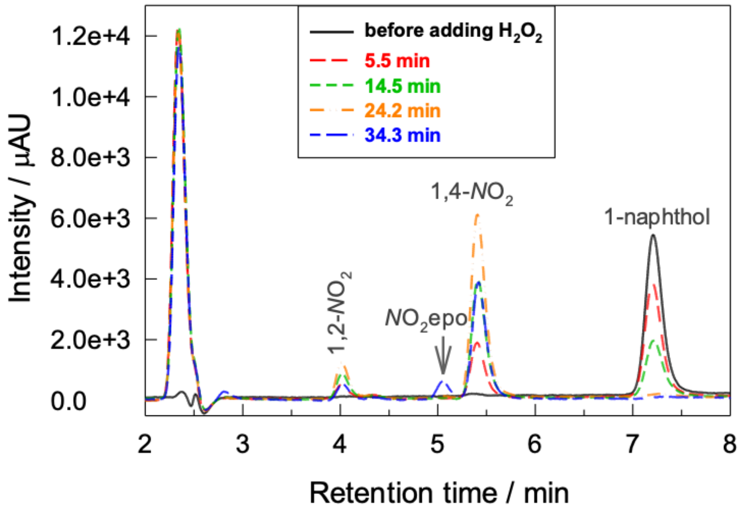

Figure S3. Dynamics of generation of 1,2-*N*O_2, 1,4-NO_2 and *N*O_2epo alongside with the 1-naphthol collapse. Conditions: [1-naphthol] 1.1 $\times 10^{-4}$ M, [**1**] 1.6 $\times 10^{-7}$ M, [H_2O_2] 3.0 $\times 10^{-3}$ M, pH 6.9 (0.01 M phosphate), 25 ^{o}C, mobile phase- 60:40 (pH 3 (0.01 M phosphate) : acetonitrile), 0.5 mLmin^{-1} flow rate, wavelength of detection: 250 nm.

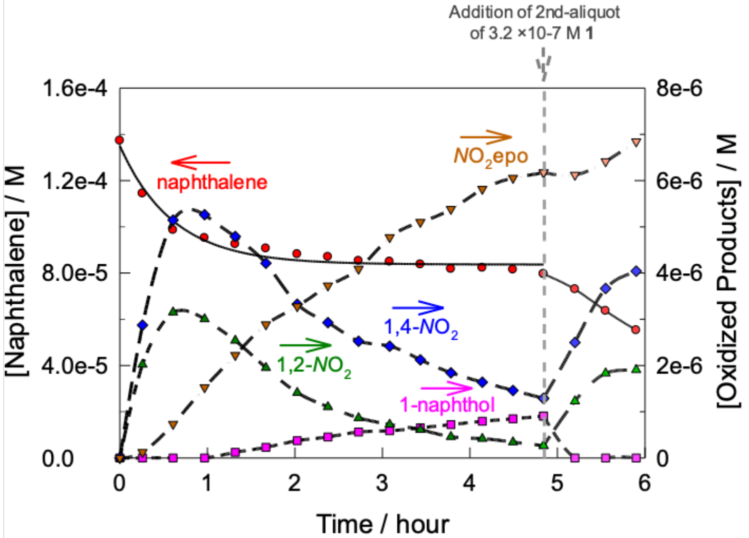

Figure S4. Dynamics of generation of 1,2-*N*O_2, 1,4-*N*O_2, *N*O_2epo and 1-naphthol alongside with naphthalene collapse. Conditions: [naphthalene] 1.4 $\times 10^{-4}$ M, [**1**] 3.2 $\times 10^{-7}$ M, [H_2O_2] 6.0 $\times 10^{-3}$ M, pH 6.9 (0.01 M phosphate), 25 ^{o}C. The second aliquot of **1** was added after 4.9 h.

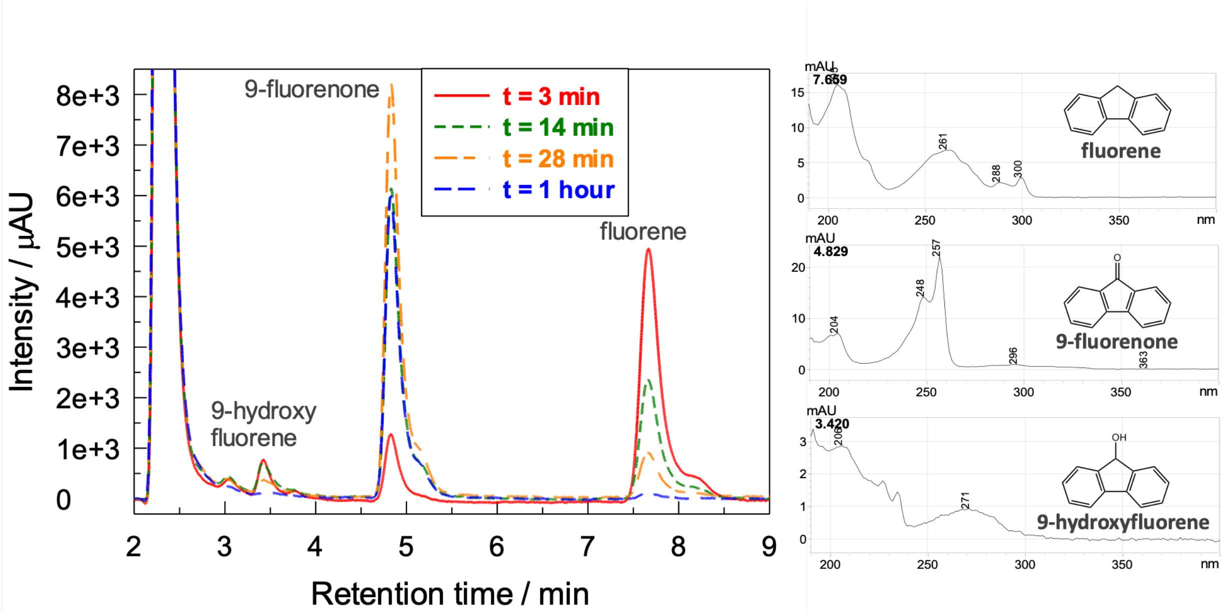

Figure S5. Time-dependent HPLC chromatograms accompanying the oxidation of fluorene. Conditions: [fluorene] 4.5 $\times 10^{-6}$ M, [**1**] 1.6 $\times 10^{-7}$ M, [H_2O_2] 0.02 M, pH 6.9 (0.01 M phosphate), 25 ^{o}C. Parameters of HPLC-PDA system: composition of isocratic mobile phase: 40-60 (pH 3 (0.01 M phosphate) - acetonitrile), 0.5 mL min^{-1} flow rate, Kinetex 5 μm EVO C-18 100 Å column (100 × 4.6 mm), wavelength of detection 261 nm. The UV-Vis spectra of fluorene and its major oxidized products detected by photodiode array (PDA) detector are shown on the right.

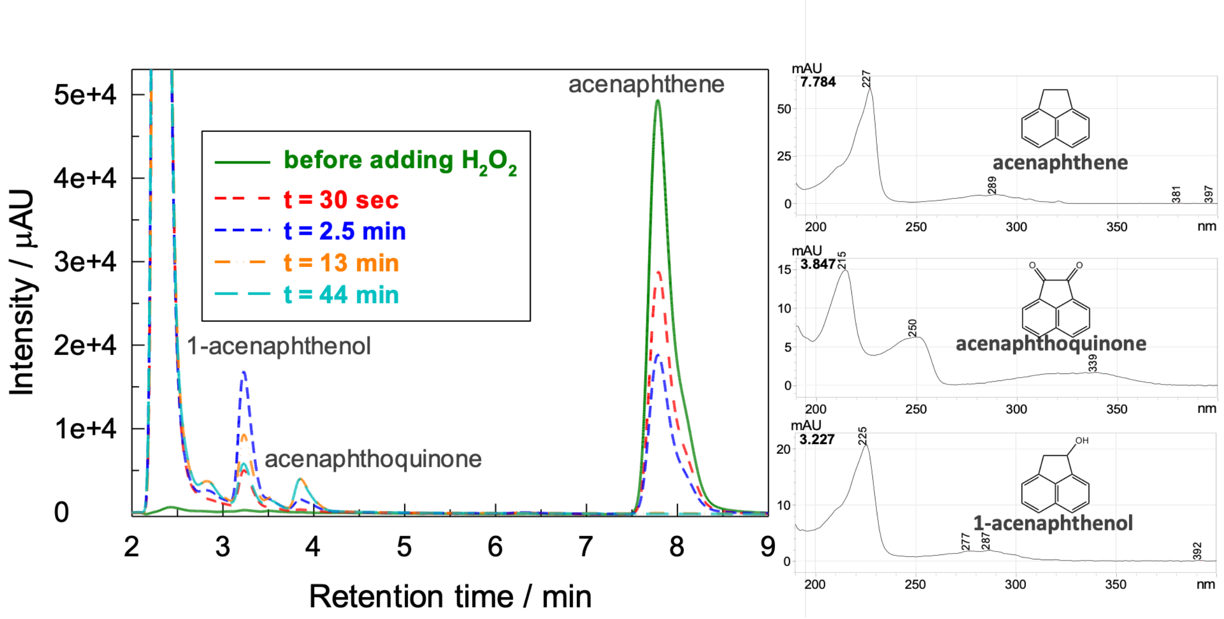

Figure S6. Time-dependent HPLC chromatograms accompanying the oxidation of acenaphthene. Conditions: [acenaphthene] 1.6×10^{-5} M, [**1**] 8.0×10^{-8} M, [H_2O_2] 0.02 M, pH 6.9 (0.01 M phosphate), 25 °C. Parameters of HPLC-PDA system: composition of isocratic mobile phase: 40-60 (pH 3 (0.01 M phosphate) - acetonitrile), 0.5 mL min^{-1} flow rate, Kinetex 5 µm EVO C-18 100 Å column (100 × 4.6 mm), wavelength of detection 227 nm. The UV-Vis spectra of acenaphthene and its major oxidized products detected by photodiode array (PDA) detector are shown on the right.

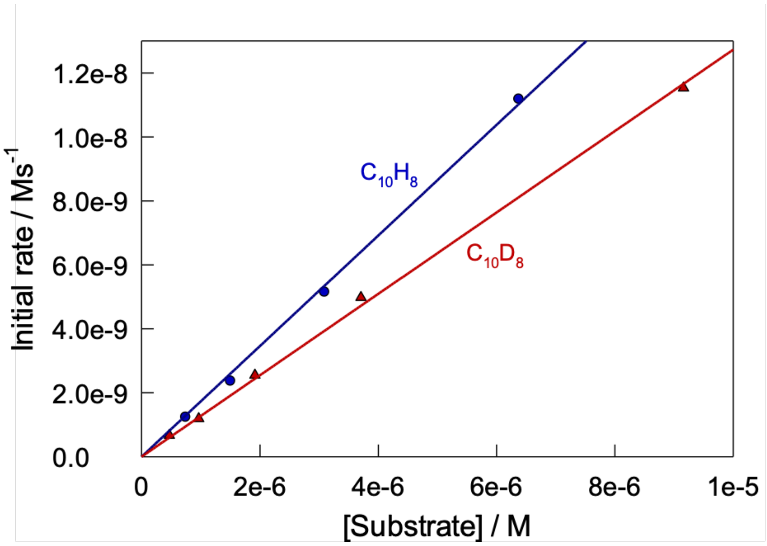

Figure S7. Comparison of initial rates of oxidation of naphthalene ($C_{10}H_8$) and perdeuterated naphthalene ($C_{10}D_8$). Conditions: [H_2O_2] 0.04 M, [**1**] 1.6 ×10^{-7} M, pH 6.9 (0.01 M phosphate), 25 ^{o}C.

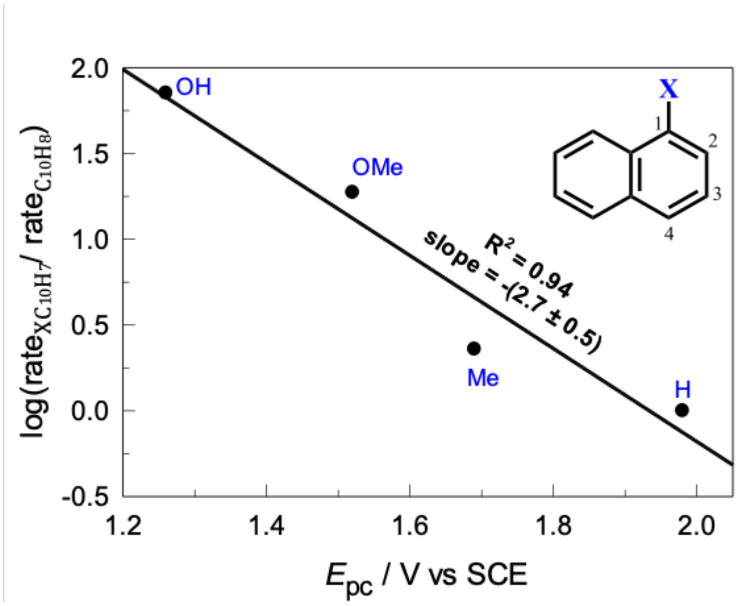

Figure S8. Relative rates of oxidation of 1-substituted naphthalenes by **1**/H_2O_2 versus E_{pc} (one-electron oxidation peak potentials) of substituted naphthalene. Conditions: [substituted naphthalene] 9.0 ×10^{-6} M, [**1**] 8.0 ×10^{-8} M, [H_2O_2] 0.01 M, pH 6.9 (0.01 M phosphate) and 25 ^{o}C.

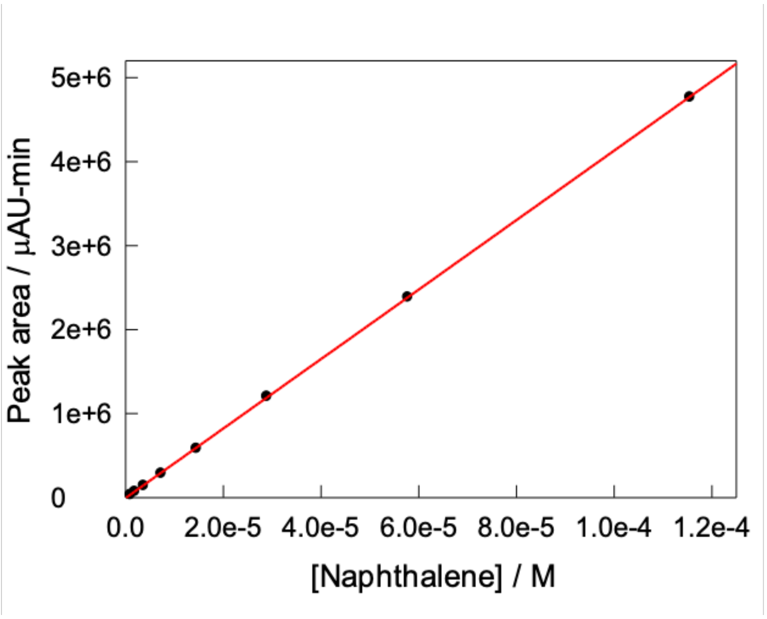

Figure S9. Naphthalene calibration curve at pH 6.9 (0.01 M phosphate) obtained by HPLC-PDA: injection volume 10 µL, mobile phase 60 : 40 acetonitrile : pH 3 (0.01 M phosphate), wavelength of detection 220 nm. The limit of detection is (2.0 ± 0.5) $\times 10^{-8}$ M.

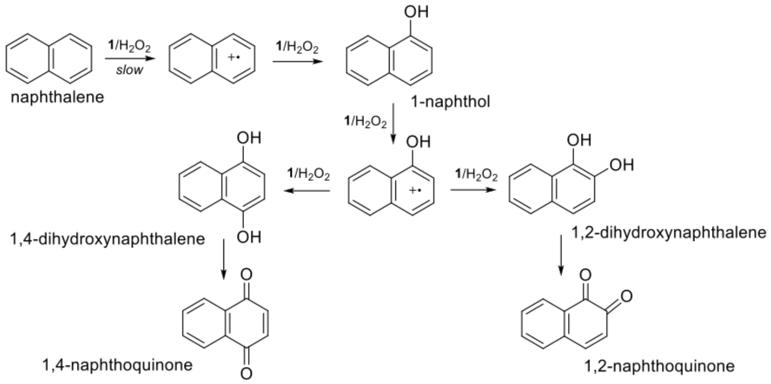

Scheme S1. Mechanism of oxidation of naphthalene by TAML/H_2O_2.
